# Remarkable Reduction in I_G_ with an Explicit Investigation of the Leakage Conduction Mechanisms in a Dual Surface-Modified Al_2_O_3_/SiO_2_ Stack Layer AlGaN/GaN MOS-HEMT

**DOI:** 10.3390/ma15249067

**Published:** 2022-12-19

**Authors:** Soumen Mazumder, Parthasarathi Pal, Kuan-Wei Lee, Yeong-Her Wang

**Affiliations:** 1 Department of Electrical Engineering, Institute of Microelectronics, National Cheng-Kung University, Tainan 701, Taiwan; 2 Department of Electronic Engineering, I-Shou University, Kaohsiung 840, Taiwan

**Keywords:** Al_2_O_3_/SiO_2_, AlGaN/GaN, MOS-HEMT, post-gate annealing (PGA)

## Abstract

We demonstrated the performance of an Al_2_O_3_/SiO_2_ stack layer AlGaN/GaN metal–oxide semiconductor (MOS) high-electron-mobility transistor (HEMT) combined with a dual surface treatment that used tetramethylammonium hydroxide (TMAH) and hydrochloric acid (HCl) with post-gate annealing (PGA) modulation at 400 °C for 10 min. A remarkable reduction in the reverse gate leakage current (I_G_) up to $1.5\times 10^{-12}\;A/\mathrm{mm}$ (@ V_G_ = −12 V) was observed in the stack layer MOS-HEMT due to the combined treatment. The performance of the dual surface-treated MOS–HEMT was significantly improved, particularly in terms of hysteresis, gate leakage, and subthreshold characteristics, with optimized gate annealing treatment. In addition, an organized gate leakage conduction mechanism in the AlGaN/GaN MOS–HEMT with the Al_2_O_3_/SiO_2_ stack gate dielectric layer was investigated before and after gate annealing treatment and compared with the conventional Schottky gate. The conduction mechanism in the reverse gate bias was Poole–Frankel emission for the Schottky-gate HEMT and the MOS–HEMT before annealing. The dominant conduction mechanism was ohmic/Poole-Frankel at low/medium forward bias. Meanwhile, gate leakage was governed by the hopping conduction mechanism in the MOS–HEMT without gate annealing modulation at a higher forward bias. After post-gate annealing (PGA) treatment, however, the leakage conduction mechanism was dominated by trap-assisted tunneling at the low to medium forward bias region and by Fowler–Nordheim tunneling at the higher forward bias region. Moreover, a decent product of maximum oscillation frequency and gate length (f_max_ × L_G_) was found to reach 27.16 GHz∙µm for the stack layer MOS–HEMT with PGA modulation. The dual surface-treated Al_2_O_3_/SiO_2_ stack layer MOS–HEMT with PGA modulation exhibited decent performance with an I_DMAX_ of 720 mA/mm, a peak extrinsic transconductance (G_MMAX_) of 120 mS/mm, a threshold voltage (V_TH_) of −4.8 V, a higher I_ON_/I_OFF_ ratio of approximately $1.2\times 10^{9}$, a subthreshold swing of 82 mV/dec, and a cutoff frequency(f_t_)/maximum frequency of (f_max_) of 7.5/13.58 GHz.

## 1. Introduction

Considering the unique features of III-nitride, e.g., high carrier density (~10^13^/cm^3^), large band gap (~3.4 eV), high saturation velocity (~$2\times 10^{7}$ cm/s), and large breakdown field (>3 MV/cm), extensive research has been conducted on AlGaN/GaN high-electron-mobility transistors (HEMTs) for high-power and high-frequency applications [1,2,3]. The high-density and high-mobility two-dimensional electron gas (2DEG) generated at the AlGaN/GaN interface enables us to understand how a power-switching transistor with low ON resistance is relevant to next-generation power conversion systems [4]. However, the performance of this type of transistor is inherently limited by high gate leakage current (I_G_) due to the strong polarization-induced electric field and Schottky gate contact [5]. The high gate leakage leads to a limited gate voltage swing (GVS), reduced radio frequency (RF) performance, and breakdown voltage (V_BR_).

Moreover, Cl_2_-based inductive coupled plasma (ICP) dry etching has been extensively used to isolate devices. This process correspondingly induces trap states in a device, leading to severe gate leakage current [6]. Lee et al. reported that surface conditions, e.g., the native oxide layer on the GaN surface or ICP etching damage, also severely affect device performance [7]. Thus, the native oxide layer or ICP etching damage must be removed from the surface to improve device performance. Numerous wet chemical solutions, including sulfuric acid (H_2_SO_4_), hydrogen fluoride (HF), hydrochloric acid (HCl), potassium hydroxide (KOH), and tetramethylammonium hydroxide (TMAH), have been used to improve the surface condition of devices [8,9,10,11,12,13,14,15]. Previous reports have suggested that TMAH surface treatment prior to gate oxide deposition can effectively enhance the performance of GaN HEMT devices to a considerable extent [7].

In addition to wet surface treatment, inserting a dielectric layer or stack structure under the metal gate can effectively suppress I_G_, improve current collapse, and provide better linearity [16]. Various dielectric materials, e.g., AlN [17], SiO_2_ [18], MgCaO [19], HfO_2_ [20], Al_2_O_3_ [21], HfAlO_X_ [22], ZrO_2_ [23], and TiO_2_ [24], have been extensively investigated. Among these materials, Al_2_O_3_ is a potential candidate for fabricating a metal-oxide semiconductor (MOS)–HEMT due to its comparatively high dielectric constant (~9 eV), large bandgap (~6.5 eV) with a significant conduction band offset (~1.91 eV), and good interface with AlGaN [25,26]. However, to reduce the gate leakage current further and improve the conduction band offset, a thin layer of SiO_2_ with a larger bandgap (~9 eV) can be inserted under the Al_2_O_3_ layer [18]. Nevertheless, the interface traps between the stack gate dielectric (Al_2_O_3_/SiO_2_) and the (Al)GaN layer affect the performance of the MOS–HEMT. A previous report suggested that post-gate annealing (PGA) is an effective method for reducing the oxide defect charge, interface-trapped charge, and oxide-trapped charge [27].

To reduce I_G_ further, and thus, improve device reliability and increase GVS, the gate leakage mechanism in AlGaN/GaN-based MOS–HEMTs before and after gate annealing should be investigated. Previous reports have indicated that Poole–Frankel emission (PFE), trap-assisted tunneling (TAT), and Fowler–Nordheim tunneling (FNT) are the dominant leakage mechanisms in the atomic layer deposition (ALD) of Al_2_O_3_/AlGaN/GaN and the low-power chemical vapor deposition (CVD) of the SiNx/AlGaN/GaN MOS–HEMT [28,29]. However, they have only investigated forward bias gate leakage characteristics. Although the forward and reverse leakage mechanisms of a SiN_X_ MOS–HEMT were investigated previously in detail, the changes in the conduction mechanism after annealing were not considered [5]. To date, no report is available on the explicit investigation of the gate leakage conduction mechanism in a dual surface-treated Al_2_O_3_/SiO_2_ stack layer MOS–HEMT before and after gate annealing modulation. 

With this objective, this work demonstrated the improved device performance with a remarkable reduction in the gate leakage current of a dual surface-treated Al_2_O_3_/SiO_2_ stack layer MOS–HEMT with PGA modulation. A detailed investigation of the conduction mechanism of gate leakage current in forward and reverse biases was performed on a MOS–HEMT before and after gate annealing treatment, and the results demonstrated how the dominant conduction mechanism was changed after PGA treatment. The dual surface-treated Al_2_O_3_/SiO_2_ stack layer MOS–HEMT with PGA modulation exhibited decent performance with a maximum drain current (I_DMAX_) of 720 mA/mm, a peak extrinsic transconductance (G_MMAX_)of 120 mS/mm, a threshold voltage (V_TH_) of −4.8 V, a higher I_ON_/I_OFF_ ratio of approximately $1.2\times 10^{9}$, a subthreshold swing (SS) of 82 mV/dec, and a cutoff frequency (f_t_)/maximum frequency (f_max_) of 7.5/13.58 GHz, with the lowest gate leakage current of $1.5\times 10^{-12}\;A/\mathrm{mm}$ and a decent (f_max_ × L_G_) of 27.16 GHz∙µm.

## 2. Materials and Methods

The AlGaN/GaN epitaxy was grown using a low-pressure metal-organic chemical vapour deposition (MOCVD) system on a p-type low-resistive (111) Si substrate. The epilayers consisted 3.9 µm GaN buffer layer, a 300 nm undoped GaN layer, a 20 nm Al_0.25_Ga_0.75_N barrier layer, and a 2 nm GaN cap layer. The measured sheet carrier concentration and Hall mobility were 6.15$\;\times \;10^{12}/{\mathrm{cm}}^{2}$ and 2338 ${\mathrm{cm}}^{2}/V\cdot s$, respectively.

**Figure 1:**
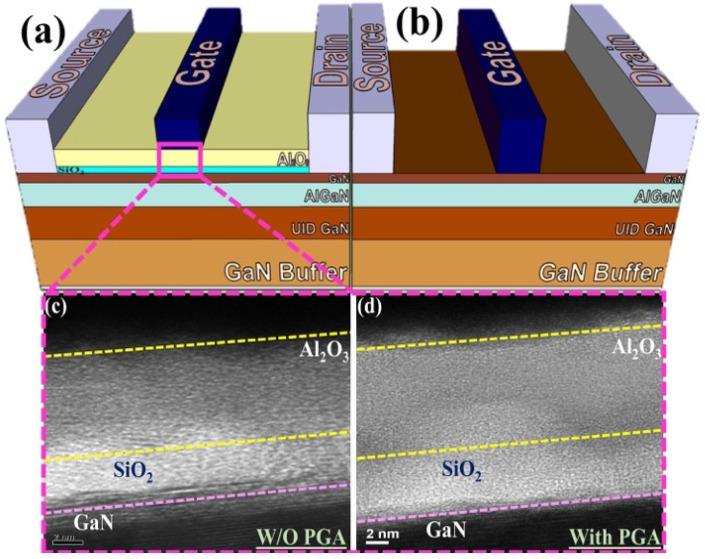
Schematic of (**a**) Al_2_O_3_/SiO_2_ MOS–HEMT and (**b**) conventional HEMT. TEM images of the MOS–HEMT (**c**) without gate annealing and (**d**) with gate annealing treatment.

Device processing started with mesa isolation by using an ICP reactive ion etching system with a Cl_2_/BCl_3_ gas mixture. Then, the sample was immersed into 5% TMAH solution at 85 °C for 1 min to remove native oxide and ICP etching damage. Thereafter, the source and the drain regions were defined via ultraviolet (UV) photolithography. Then, Ti/Al/Ni/Au (25/150/30/120 nm) metal contacts were deposited using an electron beam (e-beam) evaporator system, followed by rapid thermal annealing at 875 °C for 30 s under N_2_ ambient atmosphere to ensure good ohmic contact. Subsequently, HCl wet treatment was performed for 3 min prior to gate metal deposition for conventional HEMT and gate oxide deposition for MOS-HEMT. Then, a stack gate dielectric layer composed of a 5 nm SiO_2_ followed by a 10 nm Al_2_O_3_ layer, was deposited using an ALD system (Picosun) at 250 °C. Finally, the gate region was defined via UV photolithography, and a Ni/Au (80/100 nm) gate stack was deposited using an e-beam evaporator followed by a liftoff process. To improve device performance further, PGA was performed at 400 °C for 10 min. Sheet resistance was 434 Ω/□. For reference, a MOS–HEMT with 5 nm SiO_2_ gate dielectric and a conventional HEMT were also fabricated. The gate width (W_G_) and gate length (L_G_) were 100 µm and 2 µm for all devices, and L_GD_ and L_SG_ were both 2 µm. To understand the gate annealing treatment on the MOS–HEMT and the conventional HEMT, all devices were fabricated following the same processing conditions without PGA treatment. Figure 1a,b shows the typical schematic of a MOS–HEMT and a planar HEMT. UV photolithography was performed using an MJB3 Karl Suss mask aligner system. DC I–V and RF performance were measured with a B1500A semiconductor characterization system and an Agilent N5245A network analyzer with an HP 4142B DC monitor, respectively. To understand the gate dielectric thickness we used transmission electron microscopy (TEM) (JEOL JEM-2100F) system. After the focused ion beam, we used carbon lacey grid for better resolution of the TEM image. To understand the quantitative analysis of the surface composition and material elemental composition we did X-ray photoelectron spectroscopy (XPS) (JEOL). To analyze the effect of TMAH wet surface treatment on the performance of the stack layer MOS–HEMT device, XPS was conducted using a k-alpha X-ray photoelectron spectrometer. To stick the sample on the holder for XPS a copper foil conductive has been used. A monochromatic Al Kα X-ray source with 90° taken off-angle was used. The sputtering depth was approximately 30–50 nm.

## 3. Results

**Figure 2:**
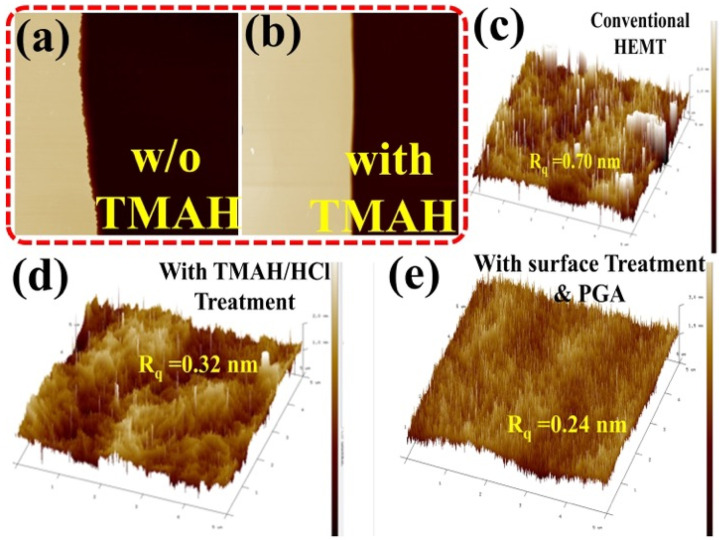
AFM profile of AlGaN/GaN device after ICP mesa etching (**a**) without and (**b**) with TMAH treatment. The 3D AFM images of the device (**c**) without, (**d**) with dual surface treatment and gate annealing modulation.

Figure 1c,d shows the TEM image of the Al_2_O_3_/SiO_2_ MOS–HEMT before and after gate annealing treatment. Clear layers of the 5 nm SiO_2_ and 10 nm Al_2_O_3_ were found without intermixing before PGA treatment. Owing to the diffusion of atoms, a less layered structure was observed after gate annealing treatment. Typical atomic force microscopy (AFM) images are shown in Figure 2a–e under different conditions. As shown in Figure 2a,b, side wall surface morphology was improved with TMAH surface treatment. After dual surface treatment with PGA modification, the root-mean-square roughness was significantly improved from 0.70 nm to 0.24 nm, subsequently enhancing device performance.

**Figure 3:**
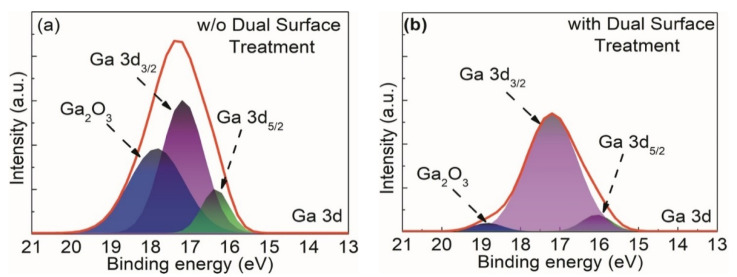
XPS of the AlGaN/GaN device (**a**) without and (**b**) with dual surface treatment.

Figure 3 illustrates the change in the atomic composition of the Ga 3 core levels before and after surface treatment, with both spectra deconvoluted into two peaks of Ga-N and Ga-O. The Ga 3d_5/2_ and Ga_2_O_3_ (Ga^3+^) peaks were de-convoluted by considering spin-orbital splitting [30]. Figure 3 clearly shows that the intensity of Ga-O is considerably lower after TMAH surface treatment. The peak intensity ratio of Ga-O/Ga-N significantly decreased to 6.6% from 63%. The removal of native oxide at the GaN surface via wet surface treatment reduced the intensity of the Ga-O bond, subsequently improving device performance as previously reported [31,32,33,34].

**Figure 4:**
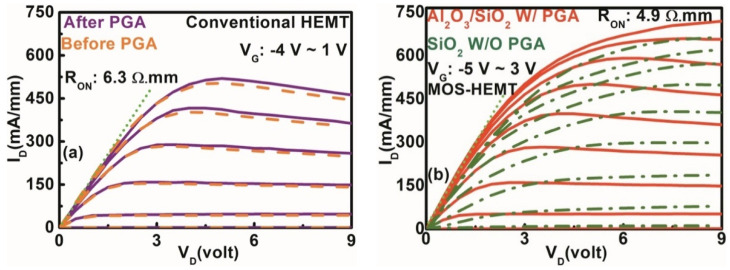
I_D_–V_D_ characteristics of (**a**) conventional HEMT with and without gate annealing and (**b**) Al_2_O_3_/SiO_2_ MOS–HEMT with and without gate annealing treatment and SiO_2_ MOS–HEMT without PGA treatment.

The typical drain current versus voltage (I_D_–V_D_) characteristics of the conventional HEMT and the Al_2_O_3_/SiO_2_ stack layer MOS–HEMT are shown in Figure 4. The I_DMAX_ of the dual surface-treated stack layer (SiO_2_) MOS–HEMT and the conventional HEMT was 720 mA/mm (650 mA/mm) (@ V_G_ = 3 V) and 520 mA/mm (@ V_G_ = 1 V), respectively, with PGA modulation, as shown in Figure 4. Better pinch-off behavior in the stack layer MOS–HEMT suggested better gate controllability than in the SiO_2_ MOS–HEMT. The conventional HEMT was not biased with higher V_G_ due to the large late leakage current. The reduction of I_DMAX_ in the conventional HEMT was attributed to the large I_G_ [35]. Moreover, I_DMAX_ was 650 mA/mm (500 mA/mm) for the MOS–HEMT (HEMT) without annealing modulation. In addition, the ON resistance (R_ON_) was significantly reduced from 6.3 Ω.mm to 4.9 Ω.mm in the MOS–HEMT due to dual surface treatment with the application of a stack dielectric layer.

**Figure 5:**
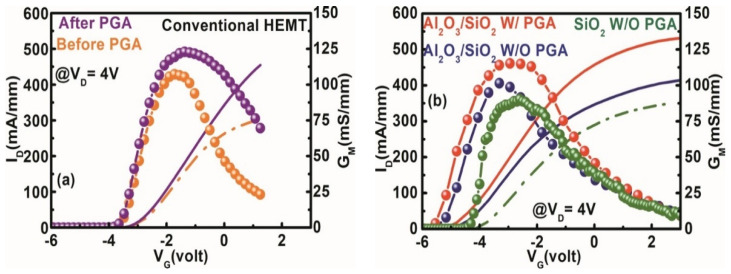
I_D_–V_G_ characteristics of (**a**) conventional HEMT with and without gate annealing and (**b**) Al_2_O_3_/SiO_2_ MOS–HEMT with and without gate annealing treatment, and SiO_2_ MOS–HEMT without PGA treatment.

The transfer characteristics of the stack layer MOS–HEMT and the conventional HEMT before and after gate annealing treatment (@ V_D_ = 4 V) are shown in Figure 5. The Al_2_O_3_/SiO_2_ stack layer MOS–HEMT exhibited a V_TH_ of −4.8 V (−4.4 V) with (without) gate annealing modulation. For the conventional HEMT and the SiO_2_ MOS–HEMT, V_TH_ was −2.7 V and −3.6 V, respectively. The threshold voltage is defined as the gate bias intercept point of the linear extrapolation of the drain current at G_MMAX_ [22]. The V_TH_ difference between HEMT and MOS–HEMT can be expressed as [5]:

(1) $$V_{\mathrm{TH},\mathrm{MOS}–\mathrm{HEMT}}\;-\;V_{\mathrm{TH},\mathrm{HEMT}}\;=-\left(\frac{Q_{int}}{\varepsilon_{ox}}\right)\cdot t_{ox}-\left(\frac{qn_{ox}}{2\varepsilon_{ox}}\right)\cdot t_{ox}^{2},$$

where *Q_int_* is the total interface charge, *t_ox_* is the thickness of the dielectric layer, $\varepsilon_{ox}$ is the effective dielectric constant of the stack dielectric layer, and *n_ox_* is the oxide bulk charge. In accordance with Equation (1), the negative shift of V_TH_ is attributable to the increment of the interface fixed charge at the interface and oxide layers and the increase in the 2DEG concentration after passivation [36,37,38]. Moreover, the increase in separation between the gate and the channel layer may be another reason for the negative shifting of V_TH_. The shift of V_TH_ to the reverse direction with gate annealing treatment was confirmed in Figure 5b.

An improvement in peak extrinsic transconductance (G_MMAX_) was observed in the dual surface-treated Al_2_O_3_/SiO_2_ stack layer MOS–HEMT after gate annealing modulation compared with the SiO_2_ MOS–HEMT or the conventional HEMT shown in Figure 5. The G_MMAX_ values were 120 mS/mm (102 mS/mm) and 123 mS/mm (110 mS/mm) in the stack layer MOS–HEMT and the conventional HEMT with annealing (without annealing) modulation. The insertion of the two gate dielectrics increased the distance between the gate and the 2DEG channel, reducing gate controllability and decreasing G_MMAX_ in MOS–HEMT. In addition, GVS, defined as the 10% drop in maximum transconductance, was calculated for both devices to understand the linearity behavior of the device [20]. GVS improved from 1.10 V to 1.92 V in the dual surface-treated stack layer MOS–HEMT after gate annealing treatment. Thus, low phase noise, device linearity, and wide dynamic range were improved after dual surface treatment and PGA modulation in the stack layer MOS–HEMT [39]. Moreover, G_MMAX_ was 91 mS/mm in the SiO_2_ MOS–HEMT.

**Figure 6:**
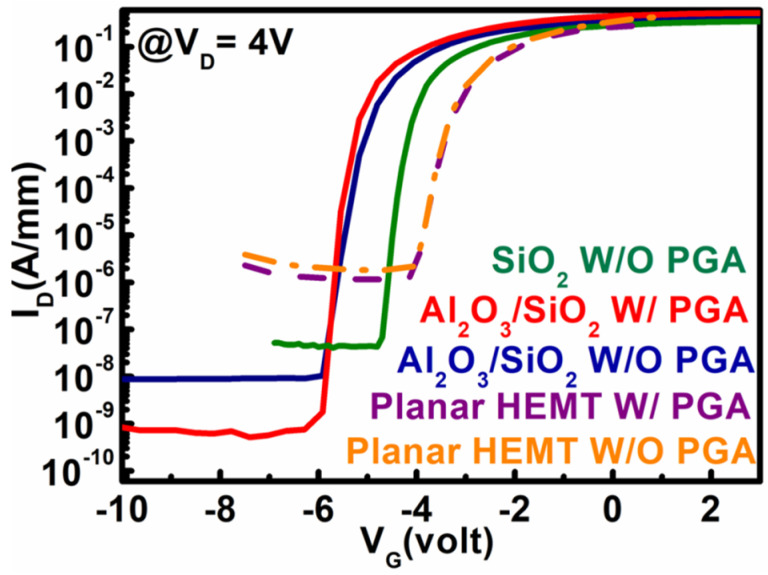
Subthreshold characteristics of conventional HEMT and Al_2_O_3_/SiO_2_ MOS–HEMT with and without gate annealing treatment and SiO_2_ MOS–HEMT without PGA treatment.

Figure 6 shows the subthreshold characteristics as a function of gate voltage (@ V_D_ = 4 V) for all devices. In this figure, the subthreshold drain leakage current was decreased by more than three orders of magnitude in the Al_2_O_3_/SiO_2_ MOS–HEMT after gate annealing modulation compared with that of the conventional HEMT. The subthreshold drain leakage current was influenced by the reverse bias gate leakage current in the pinch-off region [39]. Given that I_G_ was suppressed by the combined effects of the stack layer gate dielectric and dual surface treatment with PGA modulation in MOS–HEMT, as discussed later, the subthreshold drain leakage current was decreased to a considerable extent. Subthreshold swing (SS) also depends on I_G_. The SS values of different devices were extracted from Figure 6. The SS values were improved from 130 mV/dec to 82 mV/dec in the stack layer MOS–HEMT after gate annealing treatment. Meanwhile, for the conventional HEMT (SiO_2_ MOS–HEMT), the SS value was 178 mV/dec (91 mV/dec). The current ON/OFF (I_ON_/I_OFF_) ratios were $1.2\times 10^{9}$ and $5.8\times 10^{7}$ for the stack layer MOS–HEMT with and without PGA treatment. By contrast, no significant improvement in the current ratio was found in the planar HEMT after gate annealing treatment.

**Figure 7:**
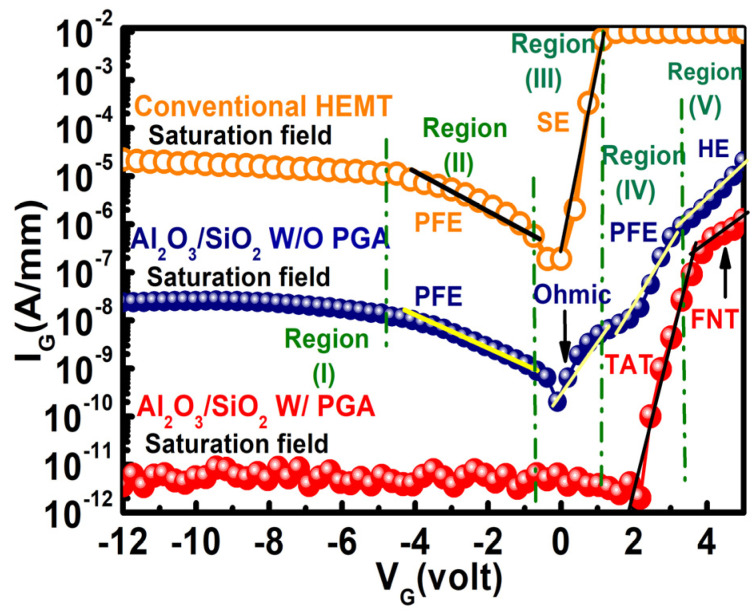
Gate leakage characteristics of the Al_2_O_3_/SiO_2_ MOS–HEMT with and without PGA treatment and conventional HEMT without PGA treatment.

The reverse and forward gate leakage current (I_G_–V_G_) characteristics of the dual surface-treated stack layer MOS–HEMT before and after gate annealing treatment and the Schottky gate HEMT without PGA were measured, and the results are presented in Figure 7. The reverse gate leakage current (@ V_G_ = −12 V) of the Al_2_O_3_/SiO_2_ MOS–HEMT was $2.3\times 10^{-8}\;A/\mathrm{mm}$ before gate annealing treatment. Evidently, I_G_ was significantly reduced by four orders of magnitude to $1.5\times 10^{-12}\;A/\mathrm{mm}$ after annealing treatment with dual surface modification. The insertion of large bandgap materials as gate dielectric combined with dual surface treatment and PGA modulation reduced I_G_ to a considerable extent.

**Figure 8:**
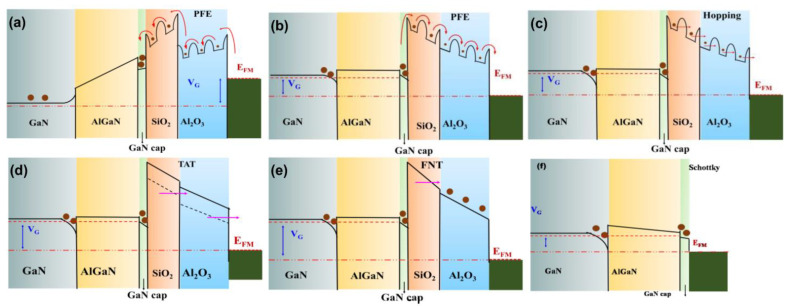
Conduction band edge diagram of Al_2_O_3_/SiO_2_ MOS–HEMT (**a**–**c**) before and (**d**), (**e**) after PGA treatment, and (**f**) conventional HEMT under different operating regions and showing the dominant conduction mechanism at each region.

To explore the charge of transportation mechanisms responsible for the gate leakage phenomenon, I_G_–V_G_ characteristics were divided into five regions as indicated in Figure 7. Leakage characteristics were analyzed in different regions to determine the dominant leakage mechanism for each particular region. The multiple conduction mechanism was studied to justify the appropriate charge transport phenomenon in the stack layer MOS–HEMT before and after gate annealing treatment and conventional HEMT. The conduction band edge diagram of the MOS–HEMT before and after PGA treatment and HEMT under different operating regions that illustrated the conduction mechanisms is shown in Figure 8.

### 3.1. *Gate Leakage Mechanisms in the AlGaN/GaN MOS–HEMT before Gate Annealing*

**Figure 9:**
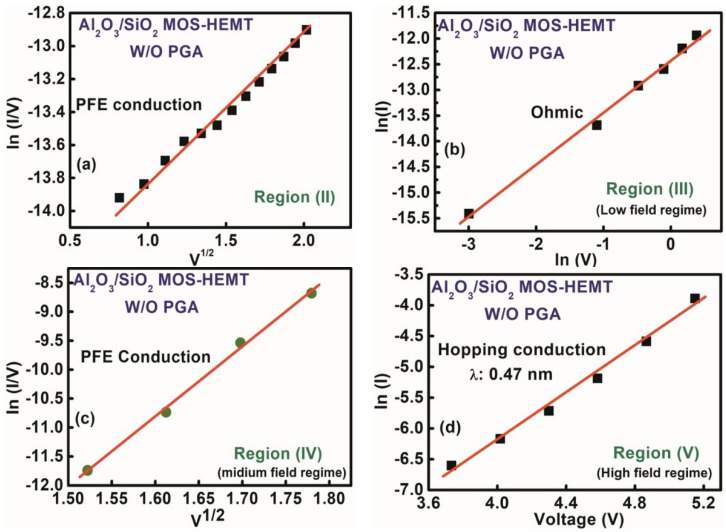
Gate leakage conduction mechanism of the Al_2_O_3_/SiO_2_ MOS–HEMT before gate annealing treatment (**a**) region II, (**b**) region III, (**c**) region IV, (**d**) region V.

For the stack layer MOS–HEMT before annealing treatment, as indicated in Region (I) for V_G_  ≤ V_TH_, the leakage current was saturated due to the saturation of the vertical electrical fields across the gate dielectric and the barrier layer [5]. However, the I_G_–V_G_ characteristics in Region (II) exhibited dependency on the applied electric field, and the PFE mechanism clearly dominated this region, as suggested in the fitted curve of [ln (I/V) vs V^1/2^] in Figure 9a. A comparatively high electric field supported the PFE conduction depicted in the fitted Figure 9a, and the charge transferred through a trap shown in the band edge diagram in Figure 8a in this region exhibited the following relation [5,40]:

(2) $$\;\ln\left(\frac{J_{PFE}}{E_{Di}}\right)=m\sqrt{E_{Di}}+c$$

(3) $$m=\frac{q}{kT}\sqrt{\frac{q}{\pi \varepsilon_{Di}}}$$

where $\varepsilon_{Di}$ is the permittivity of the dielectric materials, *k_B_* is the Boltzmann’s constant, *T* is the temperature, and *q* is the electronic charge. The effective dielectric constant (*ɛ_Di_*) was extracted to 7.2 from the ln (I/V) vs V^1/2^ characteristics, which was sufficiently close to the calculated effective dielectric constant of the Al_2_O_3_/SiO_2_ layer [41].

The dominant leakage conduction mechanism in Region (III) was ohmic due to the linear relationship of ln (I) vs. ln (V), with a slope value close to 1, as shown in Figure 9b. The leakage mechanism was assumed to be PFE at a comparatively higher voltage region. The linear fitting in Figure 9c further confirmed PFE conduction because electrons can be de-trapped with an increased electric field as shown in the band edge diagram in Figure 8b. In addition, the dominant conduction mechanism in a high field region (V_G_ ≥ 3 V) was satisfied with hopping conduction from the fitted curve of ln (I) vs. V, as shown in Figure 9d. Hopping distance (λ) can be extracted from the fitted curve by considering the following equation [40,42]:

(4) $$J=qn\lambda \;exp\left(\frac{q\lambda E-E_{a}}{kT}\right)$$

where *n* is the electron concentration, *f* is the thermal vibration frequency of the trapping sites, *E* is the corresponding electric field, and *E_a_* is the activation energy. The hopping distance was calibrated to 0.47 nm by using Equation (4). The electrons can overcome the hopping distance (λ) in the higher field region, as shown in Figure 8c, due to the higher energy.

### 3.2. *Gate Leakage Mechanisms in the AlGaN/GaN MOS–HEMT after Gate Annealing*

**Figure 10:**
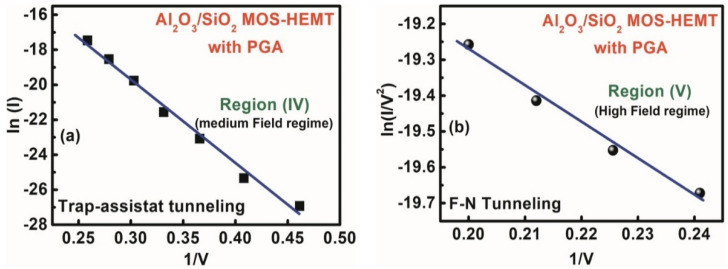
Gate leakage conduction mechanism of the Al_2_O_3_/SiO_2_ MOS–HEMT after gate annealing treatment (**a**) region IV, (**b**) region V.

The leakage mechanism of the Al_2_O_3_/SiO_2_ MOS–HEMT after gate annealing modulation was also investigated as shown in Figure 10. After gate annealing modulation, shallow traps were reduced [39] and I_G_ was independent of gate voltage at V_G_ ≤ 2 V due to the saturation of the electric field [5]. The conduction band diagram in Region (IV) for the stack layer MOS–HEMT after annealing treatment is shown in Figure 8d. In this region, leakage transportation was estimated from the fitting curve of [ln (I) vs. 1/V], as shown in Figure 10a, to a two-step TAT mechanism. The electric field dependence of the TAT current (*J_TAT_*) is given by the following equation [43]:

(5) $$\;J_{TAT}=Aexp\left(\frac{-8\pi \sqrt{2qm^{*}\emptyset_{T}^{3}}}{3hE}\right)$$

where $\emptyset_{T}$ is the trapped energy of electron traps with respect to the conduction band edge, *A* is a constant, and *h* is Planck’s constant. Shallow traps were reduced through the creation of deep traps via PGA modulation, causing the conduction mechanism to shift toward TAT from PFE after gate annealing treatment as indicated in the band diagram of TAT in Figure 8d [44].

Consequently, the leakage mechanism in the high forward bias region was dominated by FNT across the Al_2_O_3_/SiO_2_ gate dielectric layer, as shown in Figure 10b. FNT current density (*J_FNT_*) can be related to the electric field across the dielectric (*E_Di_*) by [45]:

(6) $$\ln\left(\frac{J_{FNT}}{E_{Di}^{2}}\right)=ln\;\left(A^{\prime }\right)-\frac{B\prime }{\left|E_{Di}\right|}$$

where $A^{\prime }$ is a constant, $B^{\prime }$ = 8π (2 m_n_*(q$\emptyset_{eff}$)^3^)^1/2^/(3qh), m_n_* is the effective mass of the electron in the gate dielectric, $\emptyset_{eff}$ is the effective barrier height of the electrons for FNT, and *h* is Planck’s constant. The linear relationship of the $\ln\left(\frac{I}{V^{2}}\right)\;\mathrm{vs}.\;1/V$ graph in Figure 10b verified the FNT conduction mechanism in Region (V) for the MOS–HEMT after gate annealing modulation. The band edge diagram of FNT is shown in Figure 8e.

### 3.3. *Gate Leakage Mechanisms in the AlGaN/GaN HEMT*

**Figure 11:**
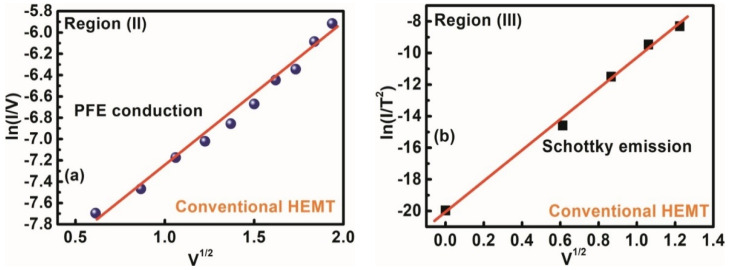
Gate leakage conduction mechanism of the conventional HEMT (**a**) region II, (**b**) region III.

We also investigated the conduction mechanism of leakage current in the conventional HEMT without PGA by dividing the I_G_–V_G_ characteristics into different regions, as shown in Figure 7. From Figure 7, I_G_ (@ V_G_ ≤ −4 V) was clearly saturated due to the saturation of the vertical electrical fields as mentioned previously. The conduction mechanism in Region (II) was confirmed as PFE conduction from the ln (I/V) vs. V^1/2^ graph by following Equation (2), similar to the stack layer MOS–HEMT before PGA treatment, as shown in Figure 11a. In addition, Schottky emission (SE) dominated Region (III) with increasing electric fields at V_G_ > 0 from the linear slope of ln (I/T^2^) vs. V^1/2^, as shown in Figure 11b, in accordance with the following relation [7]:

(7) $$I_{G}=A*T^{2}exp\left(\frac{-\emptyset_{B}}{kT}\right)exp\left(\frac{S_{SE}\sqrt{V}}{kT\sqrt{d}}\right)$$

(8) SSE=12(q3πε0εr)12

where $S_{SE}$ is the SE lowering coefficients, and $\emptyset_{B}$ is the Schottky barrier height as depicted in the conduction band edge of Region (III) in Figure 8f. In general, SE leads to conduction through the contact interface rather than from bulk material. By contrast, PFE is closely related to the tunneling of carriers and associated with the wide distribution of traps in the band gap of dielectric materials, which originates from impurities and/or structural defects.

**Figure 12:**
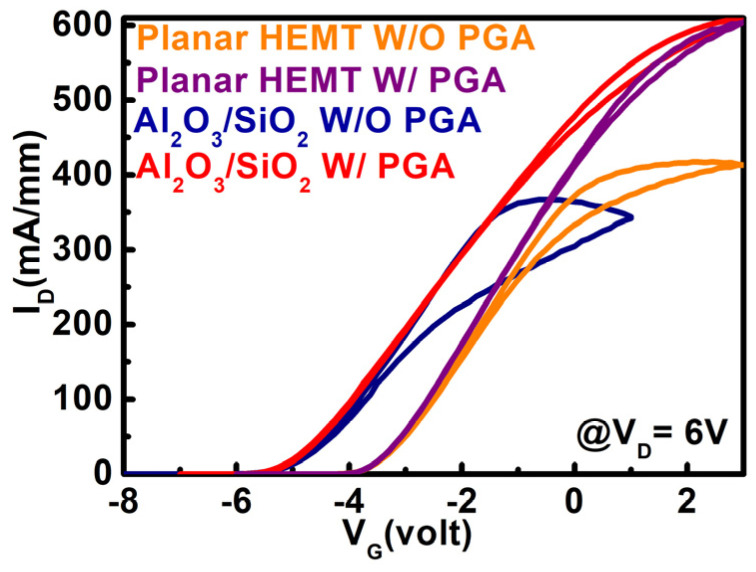
Hysteresis characteristics of the conventional HEMT and Al_2_O_3_/SiO_2_ MOS–HEMT with and without PGA treatment.

To understand the interface quality of the devices, the hysteresis characteristics of the Al_2_O_3_/SiO_2_ stack layer MOS–HEMT and the conventional MOS–HEMT were measured (@ V_G_ = 6 V) before and after gate annealing treatment, as shown in Figure 12. Hysteresis behavior was significantly improved after gate annealing modulation in both devices. After gate annealing modulation, the MOS–HEMT exhibited nearly low hysteresis of 0.1 V due to the affective neutralization of the surface caused by the combined effects of TMAH/HCl surface treatment with gate annealing modulation [7]. In addition, a counterclockwise hysteresis was observed in both devices. No surface states were available to capture electrons at a high gate voltage due to the presence of acceptor-like surface states, and electron density in the 2DEG channel was increased to raise the channel current, resulting in counterclockwise hysteresis [7,46].

**Figure 13:**
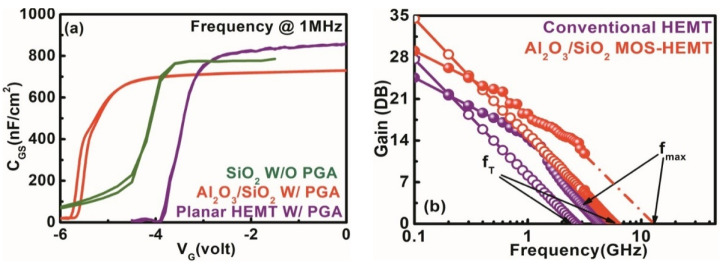
(**a**) Capacitance voltage characteristics of the conventional HEMT and the Al_2_O_3_/SiO_2_ MOS–HEMT with gate annealing treatment and SiO_2_ MOS–HEMT without PGA treatment. (**b**) High-frequency characteristics of stack layer MOS–HEMT and conventional HEMT.

To understand the reduction of trap states after dual surface treatment and PGA modulation with the stack layer gate dielectric in the MOS–HEMT compared with that in the conventional HEMT, capacitance-voltage (C–V) measurements were performed at 1 MHz for both devices as shown in Figure 13a. The high-frequency performance of the stack layer MOS–HEMT and the conventional HEMT without PGA, and short-circuit current gain (|H21|), maximum stable gain/maximum available gain (MSG/MAG) were measured as shown in Figure 13b. The measured cut-off frequency (ft) and maximum oscillation frequency (fmax) of the MOS–HEMT were 7.5 GHz and 13.5 GHz, while those for the conventional HEMT were only 2.7 GHz and 5 GHz, respectively. The comparatively higher (f_max_ × L_G_) was recorded in the MOS–HEMT after PGA modification in contrast with previous reports as indicated in Table 1. The interface state density (D_it_) for the dual surface-treated MOS–HEMT can be extracted from a previously reported formula [47] to be $1.61\times 10^{12\;}{\mathrm{eV}}^{-1}{\mathrm{cm}}^{-2}$, which is significantly improved from that of the conventional HEMT $\left(1.1\times 10^{13}\;{\mathrm{eV}}^{-1}{\mathrm{cm}}^{-2}\right).$ For the SiO_2_ MOS–HEMT, D_it_ was $3.8\times 10^{12}{\;\mathrm{eV}}^{-1}{\mathrm{cm}}^{-2}$. Given the combined effects of dual surface treatment and the stack dielectric layer with gate annealing modulation, D_it_ was significantly reduced up to one order of magnitude lower in the MOS–HEMT compared with that in the conventional HEMT. Table 1 presents the DC performance and high-frequency comparison of the different gate structure MOS–HEMTs, including the PGA modulated dual surface-treated Al_2_O_3_/SiO_2_ stack layer MOS–HEMT [17,19,31,48,49,50,51].

## 4. Conclusions

In summary, we successfully demonstrated the performance of an Al_2_O_3_/SiO_2_ stack layer MOS–HEMT that used TMAH and HCl dual surface treatment prior to gate oxide deposition with PGA modulation. The off-state gate leakage current was remarkably reduced to $1.5\times 10^{-12}\;A/\mathrm{mm}$, which was seven orders of magnitude lower (~10^−5^ A/mm) than that of the conventional HEMT. A significant reduction in I_G_ was observed in MOS–HEMT due to the combined effects of dual surface treatment and the stack gate dielectric layer with gate annealing modulation at 400 °C. In addition, a systematic investigation of the gate leakage conduction mechanism of the conventional HEMT and the MOS–HEMT before and after PGA modulation was conducted. At reverse bias, the PFE conduction mechanism dominated both devices. At low and medium forward bias, the dominant conduction mechanisms were ohmic and PFE, and at higher forward bias, gate leakage was governed by the hopping conduction mechanism for the MOS–HEMT before PGA. By contrast, after the gate annealing treatment of MOS–HEMT, the dominant leakage conduction mechanism was TAT at the low to medium forward bias region and FNT at the higher forward bias region due to the reduction of shallow traps. 

## Figures and Tables

**Table 1:** Comparison of DC/RF performances for different gate structure AlGaN/GaN MOS-HEMTs with dual surface treated Al_2_O_3_/SiO_2_ stack layer MOS-HEMT.

**Reference**	**L_G_ (µm)**	**Gate Dielectric (Thickness)**	**I_DMAX_** **(mA/mm)**	**G_MMAX_** **(mS/mm)**	**SS** **(mV/dec)**	**I_ON_/** **I_OFF_**	**Gate Leakage (A/mm)**	**f_MAX_ (GHz)**	**f_MAX_ × L_G_** **(GHz·µm)**
[19]	0.09 (T-gate)	Mg_0.25_Ca_0.75_O (4 nm)	1250	345 (@ V_D_ = 9 V)	104	~10^8^	~10^−7^ (@V_G_ = −7 V)	160	14.4
[27]	1 (rectangular gate)	Al_2_O_3_ (12 nm)	853.3	159 (@ V_D_ = 7 V)	90.3	~10^7^	~10^−8^ (@ V_G_ = −100 V)	23.4	23.4
[48]	1 (rectangular gate)	HfO_2_/Y_2_O_3_ (12/1 nm)	600	4.5 (@ V_D_ = 0.05 V)	70	10^9^	~10^−10^ (@ V_G_ = −9 V)	-	-
[31]	0.15 (T-gate)	Al_2_O_3_ (7 nm)	859	484 (@ V_D_ = 5 V)	-	-	~10^−8^ (@ V_G_ = −4 V)	100	15
[49]	1 (rectangular gate)	Al_2_O_3_ (20 nm)	855	140.6 (@ V_D_ = 7 V)	-	-	~10^−9^ (@ V_G_ = −50 V)	19.1	19.1
[50]	0.4 (T-gate)	TiO_2_/NiO (>35 nm)	709	149 (@ V_D_ = 10 V)	-	-	~10^−9^ (@ V_G_ = −10 V)	40	16
[51]	1 (rectangular gate)	ZrO_2_/Al_2_O_3_ (12/1 nm)	847	181 (@ V_D_ = 4 V)	95	~10^7^	-	9.1	9.1
This work	2 (rectangular gate)	Al_2_O_3_/SiO_2_ (10/5 nm)	720	120 (@ V_D_ = 4 V)	82	10^9^	10^−12^ (@ V_G_ = −12 V)	13.58	27.16

## Data Availability

The data presented in this study are available on request from the corresponding author.
